# The Impact of COVID-19 on Food Stockpiling Behavior over Time in China

**DOI:** 10.3390/foods10123076

**Published:** 2021-12-10

**Authors:** Erpeng Wang, Zhifeng Gao

**Affiliations:** 1 School of Economics and Management, Nanjing Tech University, Nanjing 211816, China; 2 Food and Resource Economics Department, University of Florida, Gainesville, FL 32611, USA

**Keywords:** COVID-19, food stockpiling behavior, China

## Abstract

Studying the impact of COVID-19 on consumer food stockpiling behavior is timely and imperative. It can provide important information and help to understand whether consumers permanently change their behavior or return to their old habits in the long run. This study analyzed Chinese consumers’ food stockpiling behavior using six rounds of nationwide surveys in China from December 2020 to July 2021. The results show that the scale of food reserves extended from 3.03 to 10.01 days after the outbreak of COVID-19, then dropped to a “new normal” plateau and kept fluctuating with the tide of the COVID-19 pandemic. Consumers who stockpile food for “Avoiding shortage” and “Pursuing ease” are going to stockpile food on a larger scale, implying a supply shock may affect the demand side. Those who perceive a higher level of severity of the pandemic are less likely to return to their old habits. Finally, although consumers’ food stockpiling behavior fluctuates with the tide of COVID-19 pandemic, it gradually returns to old habits over time.

## 1. Introduction

The up-and-down food stockpiling behavior with the changes in newly confirmed cases of COVID-19 is a great challenge for the food system and policymakers. Studying the impact of COVID-19 on food stockpiling behavior is timely and imperative. It provides additional market information and helps to understand whether the impact is just a short shock or whether it permanently affects consumer behavior. The study aimed to analyze Chinese consumers’ food stockpiling behavior during the pandemic and investigate the impact of motivation and risk perception on such behavior. Specific objectives included (1) determining the difference in COVID-19’s impact on food stockpiling behavior in eight-month periods; (2) investigating how motivation and risk perception affect consumer food stockpiling behavior over time; (3) identifying factors that have an impact on consumer stockpiling behavior.

COVID-19, the global pandemic, has caused huge economic damage with cities locked down and people sheltered at home since January 2020 [1]. As COVID-19 began to spread worldwide in 2020, it is changing consumers’ daily lives, and the world seems to have retreated into “survival mode”. On the one hand, from China to the US, many people have decreased their consumption, increased their family savings, and delayed investments and major purchases (e.g., automobiles, appliances, and travel) [2]. On the other hand, stockpiling behaviors have been reported in many countries [3]. Food and other household essentials are on the top of consumers’ shopping lists in such situations [4]. Consumers’ demand for some food items has spiked, because consumers panic about buying essential items for home consumption [5]. Some panic buying was caused by warnings of supply shocks such as meat processing facilities shutting down [6].

Behavioral change is determined by a complex set of interlinked personal and environmental factors [7]. Numerous factors associated with a person’s physical, social, and economic environment can further encourage or inhibit behavioral change [7]. Unexpected events, such as economic recession, natural disasters, and pandemics, can change behaviors at both the personal and social levels. Thinking of the consequences of 9/11 and the Great Recession of 2008, the world has changed drastically in many aspects, and some things have changed permanently.

Recently, many studies have been published on the impact of COVID-19 on consumer purchasing behavior [8,9,10]. However, limited research has examined consumer food stockpiling behavior, especially in both the short and long terms. A key question for the food industry is to what extent the increase in food stockpiling will persist after COVID-19 restrictions are lifted and when the pandemic finally diminishes [5]. Will COVID-19 drive lasting changes in consumer food stockpiling behavior? Or will consumers return to the old norm in the long term? Understanding the impact of the COVID-19 pandemic on consumer food stockpiling behavior can provide critical information for governments and policymakers to develop strategies and policies to reduce the turbulence along the supply chain such as adjusting inventory and response strategies [1].

## 2. Literature Review

Consumer stockpiling behavior has frequently been observed, where many consumers buy unusually large amounts of products to avoid possible future shortages or rising prices [11,12]. After public health officials across the world recommended social distancing and governments implemented stay-at-home orders and travel restrictions, retail food companies and farmers experienced both demand and supply shocks. Consumer expenditure on groceries during the COVID-19 pandemic increased because of the temporary closure of supermarkets and other groceries providers [13]. The change from eating out to eating at home has resulted in shortages and declining inventories of some food products at supermarkets, grocery stores, and convenience stores [14]. Consumers’ food stockpiling behavior results in some empty store shelves. Irrational hoarding leads to panic buying, which will result in food waste later and cause negative externalities to the society [12]. Food stockpiling behavior during the COVID-19 pandemic has created unprecedented challenges for the agri-food industry [15].

Hoarding and panic buying are common human reactions when normal supply is perceived to be disrupted by natural disasters such as winter storms and hurricanes [16,17]. At the beginning of the COVID-19 pandemic, stockpiling behavior was observed through excess demand for toilet paper, shelf-stable food items (frozen, canned, and dried foods; snacks; beverages), and cleaning products, which translated into out of stock items at the grocery store [18]. Previous studies show that partisanship, gender, and family all affect these behaviors. Barrios and Hochberg found that partisanship played a significant role in shaping perceptions of the risk of the new pandemic [19]. This study showed several significant differences in categorical responses with Republicans spending more at restaurants and in retail shops, which is consistent with their lower levels of concern about the virus or differential risk exposure. Households with children stockpiled more, and men stockpiled less in the early days as the virus was spreading [20].

The stocking phenomenon is a complex consumer behavior fueled by a set of multiple motivations and psychological processes [21]. Reasons for food hoarding may be both rational and irrational [4]. One reason is self-sufficiency (producing). That is, home-cooked meals replacing meals purchased at restaurants may have led to purchases of a wider variety of food products than before [15]. Due to the fact of social distancing and the economic downturn, more time was spent at home, decreasing the opportunity cost of cooking and cleaning [22]. This resulted in a surge in demand for basic food products such as flour [23]. Grain products experienced the largest surge in demand, which could be due to the storable nature of raw grain, and the increase in home production of grain-based foods such as bread and bakery items [15]. Fresh fruit, vegetables, and frozen food were also in high demand [15].

Another reason is that purchasing excess household food as a stock can serve as a self-insurance mechanism to guarantee future consumption during a period of uncertainty [22]. Scarce shopping situations often seem to have a greater value for consumers and trigger extreme behaviors [24]. News media reported that customers emptied supermarket shelves to stockpile durable goods. American household spending increased by approximately 50% overall between February 26 and March 11 [20]. Panic shopping was essentially triggered by people imitating what others do in anticipation of shortages (i.e., herd mentality), perceived scarcity, loss aversion, and mistrusts in market institutions [3,25]. The spending increase was associated with an increase in perceived uncertainty and stockpiling medical and food supplies during the fever period [26].

The psychological factors that triggered panic buying with the rapid development of the emergency epidemic are also worthy of consideration. Fears of the disease and concerns about food accessibility are the factors causing such stockpiling behavior [27]. Some consumers may be influenced by their peers. Thus, when they see others buying, they may follow, which is the so-called herd effect [1]. The social psychology literature suggests reactance theory as a prominent theory in explaining consumer decision making under conditions of scarcity [28,29], which focuses on an individual’s reaction to the loss of perceived freedom. Hence, a product’s limited availability or perceived scarcity can mean a threat to or loss of personal freedom and, therefore, may trigger psychological reactance that leads to increased attention to the unavailable good. This ultimately increases consumer motivation to obtain the alternative that soon may be inaccessible [30,31]. Perceived scarcity will lead to a new urgency to purchase among consumers.

Although numerous studies have examined whether and how people influenced by an epidemic change their behavior in the short term, few studies have focused on whether the shock can last long. An individual’s preference and behavior are likely to change with disasters and accidents [32,33]. As revealed in the literature on the endogenous formation of individual preferences, these preferences are not constant over time, and they change under some circumstances [34,35,36]. Exploring consumer food stockpiling behavior in the short- and long-term during the COVID-19 pandemic can provide more information about consumer behavior.

## 3. Methodology

### 3.1. Survey Design

The survey included questions about consumer food stockpiling behaviors, motivations, and perceptions of the COVID-19 risk (Table 1). We compared the changes in food stockpiling behavior before and after the COVID-19 pandemic. Respondents reported real food stockpiling behaviors by answering the following questions: “How many days’ fresh food reserves did you used to stockpile before COVID-19?”; “How many days’ fresh food reserves did you stockpile at the beginning of COVID-19?”; “How many days’ fresh food reserves do you stockpile now?”. The scale of food reserves included 1 day, 3 days, 5 days, one week, two weeks, three weeks, and one month. Respondents were asked to select from a list of motivations for food stockpiling including “Going out less”, “Avoiding shortage”, “Fighting against rising food prices”, and “Pursuing ease”. We also used five-point Likert scales to directly ask about respondents’ risk perceptions with the questions: “How likely do you think you are to be infected with COVID-19?” and “How severe do you think the pandemic is in China?”.

### 3.2. Model Specification

Consumers stockpile food to ensure their family’s food security, subject to budget constraints [1]. Fears of the disease and concerns about food accessibility have been factors causing stockpiling behavior [4]. The dependent variable was the scale of food reserves, which is a ratio scale by nature. Therefore, ordinary least squares (OLS) method was used to study the factors affecting consumers’ food reserves. The scale of food reserves was modeled as a function of motivation, risk perception, and demographic characteristics (i.e., age, gender, income, and education).

(1) $$reserve\_now_{i}^{*}=\beta_{0}motivation_{i}+\beta_{1}risk\_perception_{i}\;+\beta_{2}reserve\_before_{i}+\beta_{3}demo_{i}+\varepsilon_{i}$$

where $motivation_{i}$ represents what motivates respondents to stockpile food, $risk\_perception_{i}$ the respondents’ perceptions of the risk of COVID-19, $demo_{i}$ includes demographic characteristics, and $reserve\_now_{i}^{*}$ represents the scale of food reserves.

Furthermore, we estimated consumers’ propensity to return to their old habits of food stockpiling. The dependent variable was the change in the food reserves scale, computed as the current food reserves scale minus that before the COVID-19 pandemic. Considering that some respondents may have returned to their old habits, the change in the food reserves scale should be zero, and positive values indicate a lower probability that respondents would return to their pre-pandemic habits. The zero-inflated Poisson regression model is among the preferred estimation techniques applied by researchers to count data with an excess of zeros [37,38]. Two sets of coefficients are estimated in the ZIP regression: the first is the output from a logistic regression to study the probability of an individual belonging to a zero-group (returning to the old habit), while the second corresponds to the additional days of food stockpiling now compared to that before the COVID-19 pandemic. We tested for over-dispersion using a statistical technique recommended by Cameron and Trivedi [39] and found the data were over-dispersed, so a zero-inflated negative binomial (ZINB) model was used to estimate the factors affecting consumers’ behavior of returning to their old habits of food stockpiling.

## 4. Data Collection and Description

### 4.1. Data Collection

**Figure 1:**
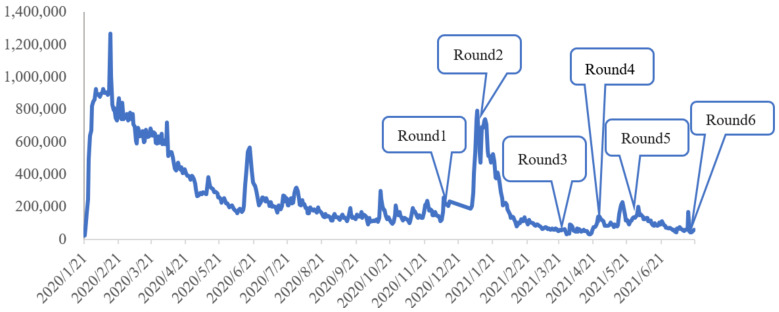
Baidu search index of the COVID-19 pandemic and 6 surveys.

Since April 2020, China has succeeded in controlling the spread of COVID-19, and only sporadic cases have been reported on the mainland, resulting in case clusters in some locations [40]. Using Baidu search data on relevant keywords, including “novel coronavirus”, “COVID-19”, and “pandemic”, we constructed a daily index by adding the Baidu index of the three search terms from https://index.baidu.com/ (accessed on 20 July 2021) to show the dynamic changes of the COVID-19 pandemic in China. Figure 1 shows that the Baidu search index of the COVID-19 pandemic kept changing with the number of newly confirmed cases. As the new wave of COVID-19 spread in China’s Jilin Province in May 2020, the Baidu search index increased significantly. Then, there was a new wave of COVID-19 cases in China’s Hebei Province in January 2021.

Six rounds of online surveys were conducted to explore the short- and long-term impact of COVID-19 on food stockpiling behavior. Just one year after the outbreak of COVID-19, we conducted the first online survey on 20–28 December 2020, then five additional rounds of surveys were conducted at intervals of approximately one or two months, on 20–23 January, 19–23 March, 20–23 April, 27–28 May, and 19–20 July in 2021 (Table 2).

### 4.2. Data Description

A professional data collection company (www.wjx.cn (accessed on 20 July 2021)) in China was hired to collect data and control the quality. The data collection company has a database of 2.6 million panel members in China. It implements several quality control measures. Firstly, all respondents needed to answer several questions about their basic personal information before entering the sample database. Secondly, at the beginning of the survey, all respondents must correctly answer some basic questions that they have responded to before. Thirdly, the IP address was validated to ensure that only one survey could be taken on one device. Finally, the questionnaire was separated into three parts, and the time spent on each part should not be less than a pre-defined minimum, because “speeding” through a survey may affect the quality and reliability of the data.

Table 3 gives the demographic statistics. We collected approximately 300–400 responses each time, with a total number of 2099 responses collected from 20 December 2020 to 20 July 2021 (Table 2). Overall, approximately 53.6% of the survey respondents were female, which is consistent with females being the primary food shoppers in China [41]. Approximately 81.90% of the respondents had a college degree, and 9.81% of the respondents had postgraduate degrees. The median monthly household income was between 12,001 and 16,000, representing the segment with the most purchasing power in China. In addition, more than 61% of the samples had children at home. Our samples can be a good representative of China’s growing middle class, which is generally defined by the National Bureau of Statistics as a family of three earning between 100,000 yuan to 500,000 yuan annually.

## 5. Results

### 5.1. Motivations of Food Stockpiling

**Figure 2:**
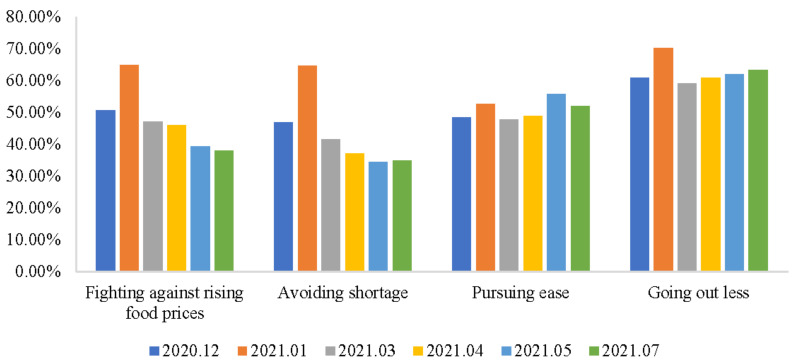
Motivations for food stockpiling.

Figure 2 reports the statistics of respondents’ motivations for food stockpiling. Motivations fueled the consumer panic buying and stocking phenomenon [21]. Identifying the changes in the motivations for food stockpiling behavior over time can help governments and food firms to understand and predict the trends in the food stockpiling phenomenon. The results in Figure 2 show that the motivations of “Fighting against rising food prices” and “Avoiding shortage” kept falling after January 2021. However, the motivation of “Pursuing ease” was still great because of the herd effect [1]. Furthermore, the motivation of “Going out less” became slightly more important as time went by because vaccinated people could also be infected if they were exposed to the Delta variant. “Going out less” became a popular and generally accepted epidemic prevention principle for Chinese consumers.

### 5.2. Consumers’ Perception of the Risk and Duration of COVID-19

**Figure 3:**
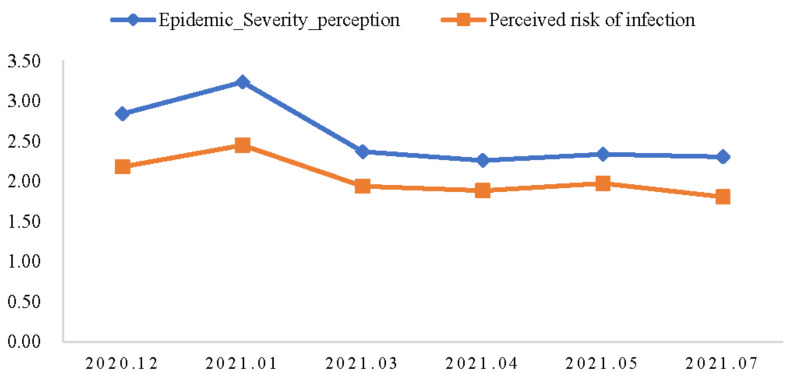
Consumers’ risk perception of the COVID-19. Perceived risk of infection: 5-point Likert scale (1—low, 5—high risk). Perceived severity of the pandemic in China: 5-point Likert scale (1—low, 5—high).

Figure 3 shows that consumers’ perceived severity of the pandemic was slightly higher than their perceived risk of infection, fluctuating with waves of the COVID-19 pandemic. The new wave of COVID-19 cases in China’s Hebei Province in January 2021 raised consumers’ risk perception of COVID-19, which then dropped to a low level. In addition, 70.03% of the respondents believed COVID-19 was highly infectious, but only 0.93% of respondents thought they were at a very high risk of the COVID-19 infection. Although most individuals overestimated the risk of a COVID-19 infection [42], this figure implies that there is a psychological pitfall of “the illusion of control” among Chinese consumers regarding COVID-19.

**Figure 4:**
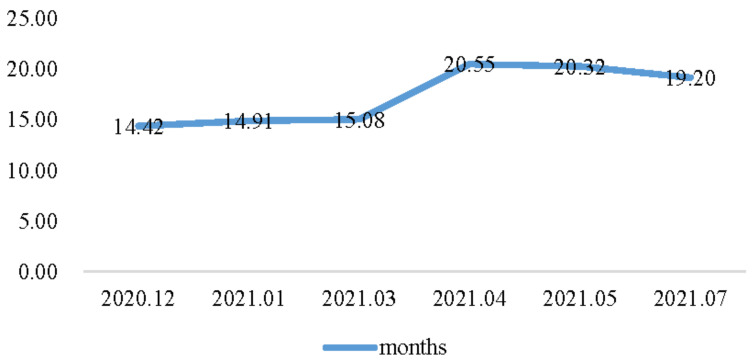
The perceived duration of COVID-19.

A previous study showed that at the beginning of the COVID-19 pandemic, the perceived duration of COVID-19 was just 2.15 months [1]. Figure 4 reports that citizens kept adjusting their expectations, up to approximately 14 months, then jumping to around 20 months. This may be because the Delta variant, which caused more infections and spread faster than the early variants, was dominant across the world1.

### 5.3. Consumer Food Stockpiling Behavior

**Figure 5:**
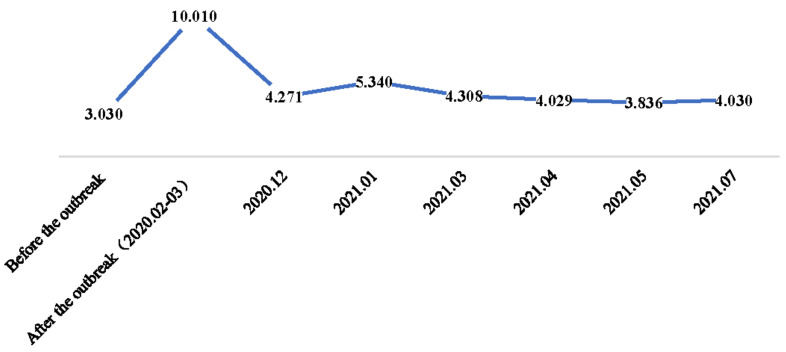
The difference in the scale of food reserves before and after the COVID-19.

Figure 5 shows that the scale of food reserves extended from 3.03 to 10.01 days after the outbreak of COVID-19, then dropped to a “new normal” plateau which was a bit higher than before. However, it kept fluctuating with the tide of the COVID-19 pandemic. After the outbreak of COVID-19, food hoarding was prevalent during the pandemic [4].

**Figure 6:**
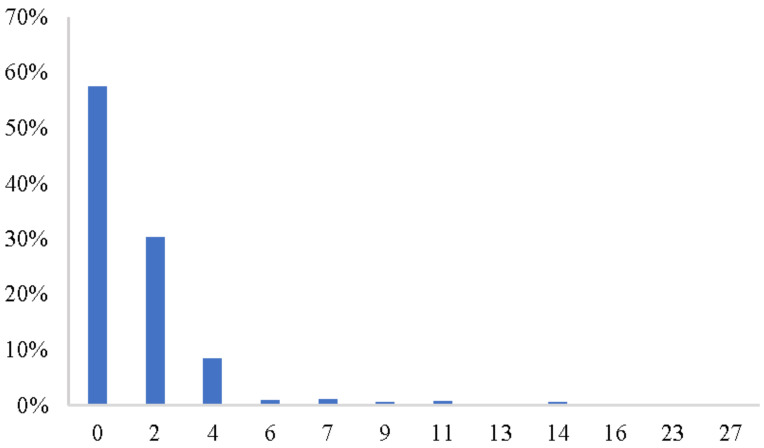
The scale change of food reserves before the COVID-19 and survey time.

If respondents return to their old habits of food stockpiling, the changes in the food reserves scale will be zero. Figure 6 shows that up until the time of the survey time, 57.42% of respondents had returned to their old habits, according to the food reserves scale, before the COVID-19 pandemic. This implies that although the lockdown and social distancing requirements during the COVID-19 pandemic have disrupted consumer buying habits in the short run [42], these habits may still have power, and many consumers may return to their old routine in the long run.

### 5.4. Factors Influencing Consumer Food Stockpiling Behavior

The OLS model was used to analyze the factors influencing consumer food stockpiling behavior with pooled data from the six rounds of surveys. The variables in Model 2 included respondents’ motivations for food stockpiling, consumer risk perceptions, and demographic variables. An additional variable, the food stockpiling habit before the outbreak, was included in Model 1. The dummy variables of survey time were also included to capture the long-run effect of the shock.

The results in Table 4 show that motivation influences behavior. Those who stockpile food for “Avoiding shortage” and “Pursuing ease” are going to stockpile food on a larger scale. As the COVID-19 pandemic let the world retreat into “survival mode” [2], “Avoiding shortage” and “Pursuing ease” were the main motivations for rational food stockpiling behavior. Panic shopping was essentially triggered by people imitating what others were doing in anticipation of shortages (i.e., herd mentality) and perceived scarcity [3,25]. Purchasing excess household food as a stock can serve as a self-insurance mechanism to guarantee future consumption during a period of uncertainty [22]. This indicates that a supply shock may affect consumers’ motivation and spill over into the demand side [43].

The results showed that the respondents who perceived a higher level of severity of the pandemic were more likely to stockpile food on a larger scale. This is consistent with a previous study that showed the impact of internal influences (e.g., fear of contagion or scarcity) on consumer shopping behavior [44]. Considering the unpredictable new wave of occasional outbreaks of COVID-19 cases, consumers’ risk perception of COVID-19 would keep changing. We are bound to see some ups and downs of food stockpiling behavior along the road to recovery.

Compared with the samples in the first-time survey (2020.12), the coefficient of survey_round 2 was positive and significant, which indicates that respondents stockpiled more food reserves when the new wave of COVID-19 cases occurred in China’s Hebei Province in January 2021. This is consistent with Dietrich’s study that showed that a spending increase was associated with a rise in perceived uncertainty and stockpiling medical and food supplies during the fever period [26]. Behavioral shifts will remain as long as uncertainty about the pandemic remains [22]. However, the coefficients of survey_round 5 and survey_round 6 became non-significant, which indicates that over time, the stockpiling started to revert to normal status, similar to that of before COVID-19.

In addition, comparing the results of Model 1 and Model 2, respondents’ food stockpiling habits before the outbreak had a significant effect on their food stockpiling behavior after COVID-19. Consumers develop habits over time about what to consume, when, and where [45]. While consumption is contextual, it is also habitual.

### 5.5. Factors Affecting the Changes in Stockpiling

A zero-inflated negative binomial regression model was applied to study the factors affecting stockpiling (Table 5). The results from the ZNIB regression model indicated that the motivations of “Avoiding shortage”, “Pursuing ease”, and “Going out less” were negatively correlated with membership in a certain zero group, implying respondents with these motivations were less likely to return to the old habit of food stockpiling. This is consistent with the fact that fears of the disease and concerns about food accessibility were factors causing such stockpiling behavior [27].

The results also show that the perceived severity of the pandemic was negatively correlated with membership in certain zero groups. This indicates that the recovery of consumer behavior from the global pandemic will be prolonged and erratic [46]. Furthermore, we took time as a continuous variable and added time square variables to explore the impact of time. These variables had no significant effect on the change in the stockpiling food scale, but their effect on membership in certain zero groups was significant. The time variable was negatively correlated with membership in the zero group, while the square of time was positively correlated with membership in the zero group. This indicates that consumer food stockpiling behavior is likely to gradually return to old habits over time. The COVID-19 pandemic may not drive lasting changes in consumer food stockpiling behavior. Females and families with children were positively correlated with the food reserves scale and negatively correlated with membership in certain zero groups, implying they are more likely to take risk aversion behavior.

## 6. Conclusions and Discussion

The lockdown and social distancing measures to combat the COVID-19 virus have generated significant disruption in consumer behavior [45]. Will consumers permanently change their behavior or will they return to their old habits once the pandemic is over? This paper identified changes in food stockpiling behavior in China with six rounds of online surveys after the COVID-19 outbreak. It showed that the COVID-19 pandemic changed Chinese families’ food stockpiling behavior, with the scale of food reserves expanding from 3.03 days to 10.01 days in the short run and then back to 4.35 days, which is a “new normal” plateau, and kept fluctuating with the tide of the COVID-19 pandemic. Considering most Chinese consumers did not like to stockpile fresh food before COVID-19, the change in consumer food stockpiling behavior during the COVID-19 pandemic poses a demand shock on the food market. Family food reserves, which can be a valuable tool for food security, also demand packaging and distribution technologies to reduce food loss and keep food fresh longer.

The results from regression analysis show that “Avoiding shortage” and “Pursuing ease” were the two main motivations for food stockpiling behavior. This implies that a supply shock in the food market may cause consumer perceived scarcity. Scarce shopping situations often trigger extreme behaviors [24], which will spill over to the demand side. In addition, considering that respondents keep adjusting food stockpiling behavior according to their risk perception of the COVID-19 pandemic, the “new normalcy” in post-pandemic consumer behavior is brittle and unstable. Behavioral shifts will remain as long as uncertainty about the pandemic remains. Therefore, food processors and distributors must make swift decisions to meet the changing demands of the COVID-19 pandemic.

The results from ZNIB regression model further show respondents with “Avoiding shortage”, “Pursuing ease”, and “Going out less” motivations are less likely to return to the old habit of food stockpiling as before the COVID-19 pandemic. Respondents with a higher level of perceived severity of the pandemic are also less likely to return to the old habit. Interestingly, the coefficients of the time variables indicated that consumers are likely to gradually return to their old habits of food stockpiling behavior in the long term. The COVID-19 pandemic may not drive lasting change in consumer food stockpiling behavior. For the industry to develop strategic plans that adapt to the changes in consumer demand, it is important to understand whether these behavioral changes will be sustained.

According to the World Health Organization (WHO), the number of COVID-19 cases and deaths has continued to decline globally [47]. How will the world be different after the COVID-19 pandemic? The story of Chinese food stockpiling behavior in the short- and long-term during the COVID-19 pandemic can provide important information for other countries. In the future, more studies should be conducted to explore whether consumers are likely to return to their old habits after the pandemic. In addition, because of the lockdown and social distancing mandates in the pandemic, it is hard for us to say that the online sample is representative of all Chinese consumers. To draw a more robust conclusion, future research can conduct similar studies covering both urban and rural areas due to the significant differences in education levels and income. It could provide more information about the influence of the pandemic on human behavior for governments and industries to develop appropriate strategies to stabilize food supply chains to avoid panic buying and to adjust the food supply according to the changes in consumer behavior.

## Figures and Tables

**Table 1:** Survey questions.

Variable	Definition
The scale of food reserves	How many days’ fresh food reserves do you stockpile now? (1 day, 3 days, 5 days, one week, two weeks, three weeks, and one month)
Motivations	What are your motivations for food stockpiling? (Fighting against rising food prices, Avoiding shortage, Pursuing ease, Going out less)
Perceived risk of infection	How likely do you think you are to be infected with COVID-19? (5-point Likert scale: 1 = low risk, 5 = high risk)
Perceived severity of pandemic	How severe do you think the pandemic is in China? (5-point Likert scale: 1 = not very severe, 5 = very severe)
Duration	How long do you think the epidemic will last from now? (number of months)
Food stockpiling habit before the pandemic	How many days’ fresh food reserves did you used to stockpile before COVID-19? (1 day, 3 days, 5 days, one week, two weeks, three weeks, and one month)

**Table 2:** Survey time and major event.

Survey	Date	Major Event Related to COVID-19
survey_round 1	20–28 December 2020	
survey_round 2	20–23 January 2021	A new wave of COVID-19 cases in China’s Hebei Province in Jan 2021.
survey_round 3	19–23 March 2021	
survey_round 4	20–23 April 2021	
survey_round 5	27–28 May 2021	
survey_round 6	19–20 July 2021	

**Table 3:** Descriptive statistics of six rounds surveys.

Sample Variable	2020.12 (*N* = 322)	2021.01 (*N* = 418)	2021.03 (*N* = 355)	2021.04 (*N* = 364)	2021.05 (*N* = 317)	2021.07 (*N* = 323)	Pooled Sample (*N* = 2099)
Gender:							
Male	41.61	49.76	44.79	45.6	48.58	47.37	46.4
Female	58.39	50.24	55.21	54.4	51.42	52.63	53.6
Age:	30.94	31	30.46	30.63	31.51	31.01	30.91
Education level:							
≤12 years	13.04	7.18	5.92	7.97	6.31	9.91	8.29
13–16 years	73.91	84.21	84.79	81.87	84.54	81.11	81.9
>16 years	13.04	8.61	9.3	10.16	9.15	8.98	9.81
Family monthly income:							
<4000 yuan	5.9	3.11	5.07	4.95	3.79	4.64	4.53
4001–8000 yuan	17.08	18.18	16.9	16.48	12.93	14.86	16.2
8001–12,000 yuan	23.29	24.4	22.82	22.53	21.14	22.6	22.87
12,001–16,000 yuan	21.12	18.42	20.56	23.63	24.61	20.74	21.39
16,001–20,000 yuan	10.87	19.86	14.08	15.38	18.61	14.55	15.72
20,001–24,000 yuan	11.49	7.89	11.27	10.44	12.3	13	10.91
≥24,001 yuan	10.25	8.13	9.3	6.59	6.62	9.6	8.38
Children under 12 years old:							
No	40.99	44.74	34.37	41.48	31.55	32.82	38.02
Yes	59.01	55.26	65.63	58.52	68.45	67.18	61.98

**Table 4:** Estimation.

Variables	Model (1)	Model (2)
Fighting against rising food prices	0.110	0.185
	(0.117)	(0.173)
Avoiding shortage	0.780 ***	0.931 ***
	(0.115)	(0.171)
Pursuing ease	0.471 ***	0.672 ***
	(0.107)	(0.159)
Going out less	0.216 *	−0.196
	(0.113)	(0.167)
Perceived severity of the pandemic	0.298 ***	0.468 ***
	(0.071)	(0.106)
Duration	0.00529	−0.00447
	(0.005)	(0.007)
Food stockpiling habit before the pandemic	1.020 ***	
	(0.022)	
Female	0.215 **	0.404 **
	(0.106)	(0.157)
Age	0.00162	−0.00852
	(0.007)	(0.010)
Education level	−0.0781	0.292
	(0.136)	(0.200)
Income	−0.0158	−0.0491
	(0.034)	(0.051)
survey_round2	0.760 ***	0.695 ***
	(0.181)	(0.267)
survey_round3	0.322 *	0.34
	(0.188)	(0.279)
survey_round4	0.349 *	0.132
	(0.188)	(0.278)
survey_round5	0.319	−0.0254
	(0.197)	(0.292)
survey_round6	0.226	0.208
	(0.196)	(0.291)
R-sq	0.58	0.078
Adjusted R-sq	0.576	0.07
AIC	7870.1	9265
BIC	7963.4	9352.8

* Significant at the 10% level. ** Significant at the 5% level. *** Significant at the 1% level.

**Table 5:** Estimation.

	Count Model		Zero Group	
Variables	Coefficient	SE	Coefficient	SE
Fighting against rising food prices	0.097	0.070	−0.053	0.140
Avoiding shortage	0.272 ***	0.071	−0.741 ***	0.139
Pursuing ease	0.029	0.067	−0.838 ***	0.134
Going out less	−0.100	0.069	−0.604 ***	0.134
Perceived severity of pandemic	0.168 ***	0.040	−0.224 ***	0.082
Duration	0.002	0.003	−0.005	0.006
Female	0.116 *	0.066	−0.093	0.127
Age	0.003	0.004	0.012	0.008
Education level	0.017	0.087	0.212	0.166
Income	−0.055 **	0.022	−0.053	0.044
Child	0.131 *	0.074	−0.228 *	0.138
Time	0.058	0.104	−0.403 **	0.188
Time × time	−0.007	0.015	0.059 **	0.026
Constant	0.165	0.328	1.913 ***	0.634

* Significant at the 10% level. ** Significant at the 5% level. *** Significant at the 1% level.

## Data Availability

Data available from authors on reasonable request.
